# Right Lung Torsion After Minimally Invasive Cardiac Surgery in a Child

**DOI:** 10.1016/j.atssr.2026.01.013

**Published:** 2026-01-29

**Authors:** Hiroki Kanamori, Akihiro Shimotakahara, Hirofumi Tomita, Yuya Komori, Kazuhiro Shoya, Satoshi Yazaki, Yukihiro Yoshimura

**Affiliations:** 1 Department of Surgery, Tokyo Metropolitan Children’s Medical Center, Fuchu, Tokyo, Japan; 2 Department of Pediatric Surgery, Fujita Health University School of Medicine, Aichi, Japan; 3 Department of Pediatric Cardiovascular Surgery, Sakakibara Heart Institute, Fuchu, Tokyo, Japan; 4 Department of Critical Care Medicine, Sakakibara Heart Institute, Fuchu, Tokyo, Japan; 5 Department of Pediatric Cardiology, Sakakibara Heart Institute, Fuchu, Tokyo, Japan; 6 Department of Cardiovascular Surgery, Tokyo Metropolitan Children’s Medical Center, Fuchu, Tokyo, Japan

## Abstract

Minimally invasive cardiac surgery (MICS) is becoming more common in pediatric congenital heart disease operation, yet its complications remain incompletely defined. We report a case of right upper and middle lobe torsion—a rare surgical complication—in a 4-year-old boy after MICS for ventricular septal defect. The torsion was discovered 1 week postoperatively while his respiratory and hemodynamic status remained relatively stable. Semiemergent resection of the twisted lobes was performed, and the patient recovered uneventfully. This case identifies lung torsion as a possible complication of MICS and illustrates the value of timely monitoring and intervention.

Lung torsion is a rare, life-threatening complication of thoracic surgery, most often reported after upper lobectomy.^1^^,^^2^ Although minimally invasive cardiac surgery (MICS) has become widely used for intracardiac repair, this complication is almost completely unrecognized. Here, we report a case of torsion of the right upper and middle lung lobes after MICS for perimembranous ventricular septal defect (VSD) in a child.

A 4-year-old boy with perimembranous VSD underwent MICS intracardiac repair through the right fourth intercostal space. He was extubated on the day of surgery. However, from postoperative day 1 to day 2, anemia progressed and the right lung field showed increased opacity on chest radiography. Computed tomography (CT) revealed reduced aeration and diminished contrast enhancement in the swollen upper and middle lobes of the right lung, so he underwent exploratory thoracotomy through the previous incision. No hematoma was found, but the right lung appeared enlarged and dark red. The cause was not identified and the chest was closed. As his condition did not improve, he was referred to our hospital.

We reevaluated the CT scan and performed bronchoscopy (Figure 1). CT demonstrated a “beak sign” of the right upper and middle lobe pulmonary artery and vein at the hilum with nonvisualization of the peripheral branches (Figure 1A). The bronchi to these lobes were severely stenotic, and the lobes were poorly aerated; the intrapulmonary CT attenuation was 40 to 50 HU, suggesting alveolar hemorrhage. Bronchoscopy showed obstruction of the right upper and middle lobe bronchi and adherence of bloody sputum (Figures 1B, 1C). These findings suggested torsion of the right upper and middle lobes.

**Figure 1:**
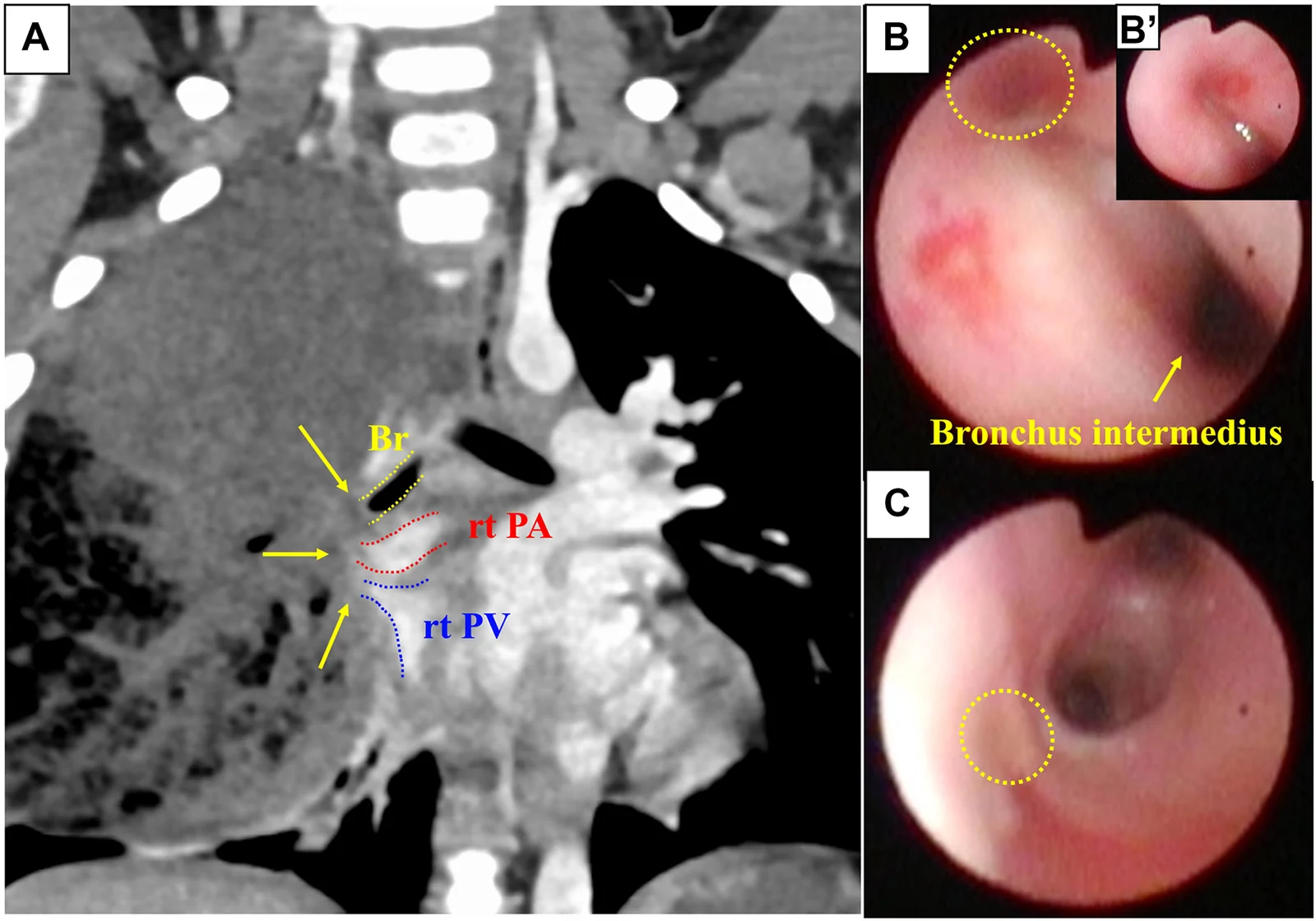
(A) Contrast-enhanced computed tomography of the chest demonstrating the “beak sign” at the right hilum involving the bronchus (Br), pulmonary artery, and pulmonary vein to the right upper and middle lobes (arrows; rt PA, right pulmonary artery; rt PV, right pulmonary vein). (B, C) Flexible bronchoscopy showing obstruction of the right upper and middle lobe bronchi indicated by circle and adherence of bloody sputum. (B′) Magnified view of the circled area in (B), showing a “fish-mouth” orifice.

Although approximately 1 week had passed since MICS—the presumed onset of lobar torsion—the patient’s respiratory and hemodynamic status remained relatively stable. To mitigate the risks of reperfusion injury and thromboembolism, the right upper and middle lobes were resected. Through median sternotomy, we first clamped the right upper pulmonary vein intrapericardially, then extended the incision laterally to an anterior thoracotomy to approach the right pleural space. The right upper and middle lobes were swollen and dark. The upper lobe was twisted 240° clockwise and the middle lobe 120° counterclockwise (Figure 2). After detorsion, both lobes were resected. The postoperative course was uneventful.

**Figure 2:**
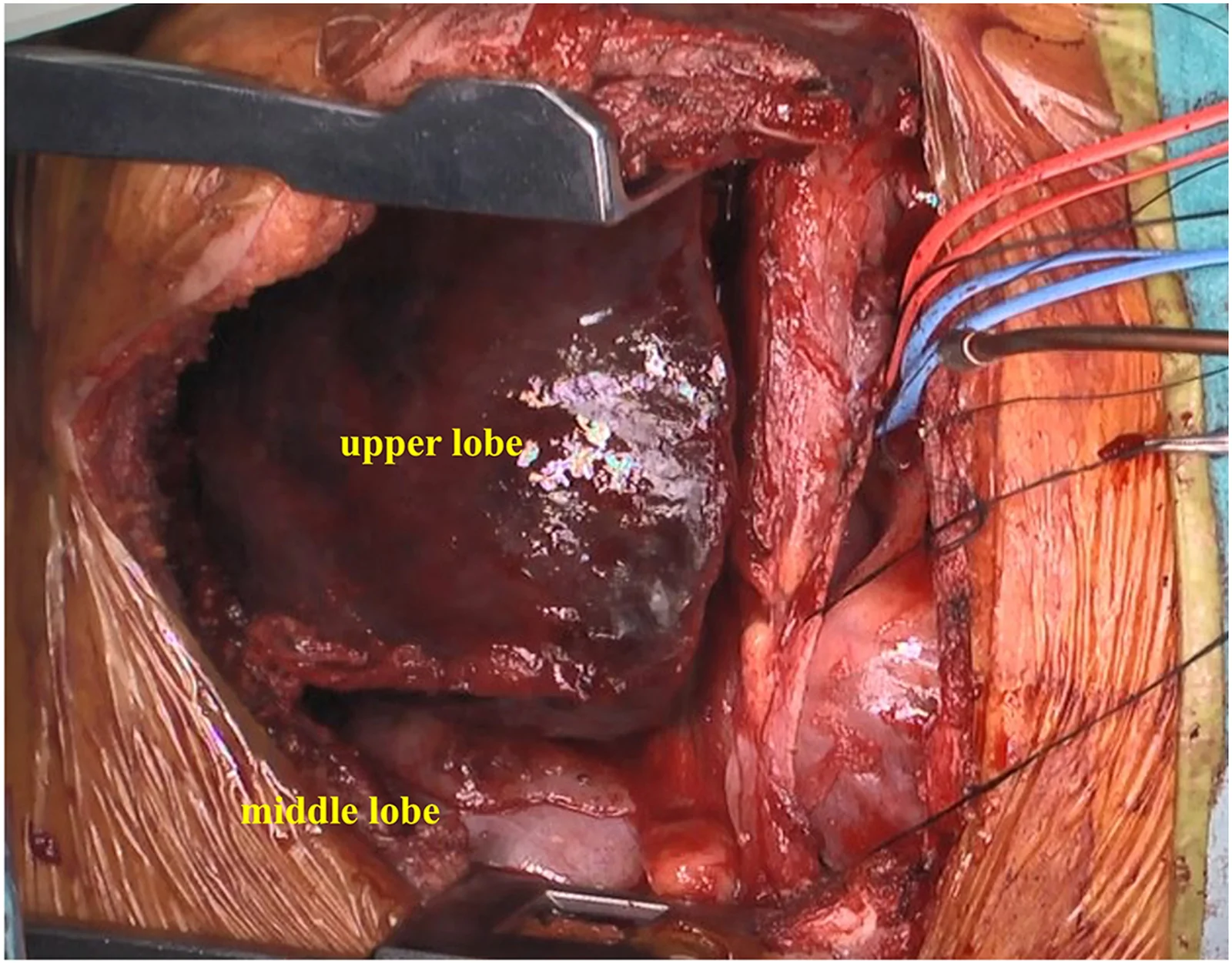
Intraoperative view showing swollen, dark-colored right upper and middle lobes. The right upper lobe is twisted ⁓240° clockwise and the middle lobe ⁓120° counterclockwise.

## Comment

MICS is increasingly used for congenital heart surgery in both pediatric and adult patients. Common complications include bleeding, unilateral pulmonary edema, stroke, and lung herniation. This case study highlights that although it is rare, lung torsion can occur after MICS. Lung torsion is defined as rotation of a pulmonary lobe around its bronchovascular pedicle, causing airway obstruction and vascular compromise.^2^ Although uncommon—reported at <0.4%,^2^ usually after upper lobectomy^1^—it is life-threatening.

We considered several factors that may have contributed to torsion during MICS for VSD in this case. Although MICS in both adults and children is more commonly approached from the anterolateral side with a skin incision parallel to the intercostal space, in this case the approach was made through a vertical incision along the right midaxillary line. This offers a cosmetic advantage because the scar is not visible from the front. However, this more dorsal incision positions the pericardiotomy posteriorly, promoting posterior collapse and compression of the right lung during normal ventilation. In addition, traction sutures on the pericardium to create the surgical field, along with instruments, may have compressed the right upper and middle lobes toward the posterior thoracic wall. We have not previously experienced lobar torsion with this approach, and the direct cause in this case remains uncertain, but this experience serves as a reminder to handle the lung gently and to try to confirm that all lobes have reexpanded before chest closure. When the small incision does not provide an adequate view of all lobes, intraoperative bronchoscopy should be considered. If bronchoscopy had shown characteristic findings—tortuous bronchus, complete airway obstruction, or a narrowed orifice^1^—torsion would probably have been realized earlier.

As an alternative surgical approach, a right anterolateral thoracotomy has also been reported for the use of MICS in VSD repair.^3^ Its advantages—easier entry, a wider window, and better midline access—may lessen pericardial suture tension and instrument pressure, reducing posterior right lung compression and torsion risk.

The major symptoms of lung torsion—dyspnea, fever, and chest pain—are nonspecific and overlap with those of pulmonary edema, a common MICS complication. Chest radiography findings (worsening lung opacity, consolidation, or collapse) are also nonspecific. In contrast, CT is highly informative when it shows bronchial or vascular malposition or abrupt stenosis. Bronchoscopy is also useful in revealing bronchial occlusion and a “fish-mouth” orifice. Once lung torsion is diagnosed or suspected, urgent surgery is essential. Dai and colleagues^1^ reported a median time to diagnosis of 4 days and a correct diagnosis rate of 57.8%, underscoring the need to consider lung torsion in the differential diagnosis.

Whether to resect the twisted lobes or to correct torsion and salvage them should be decided on a case-by-case basis; no strict guidelines exist. Some reports described cerebral infarction after detorsion due to embolism^4^ and that repositioned lobes may release inflammatory mediators, causing reperfusion injury,^5^ arguing against lobe repositioning. However, other cases have successfully salvaged lobes by detorsion without complications.^6^^,^^7^ Preoperative arterial flow and lobar viability may guide management; when preserved, early detorsion may be acceptable.^8^ In agreement, a systematic review found that when the twisted lobe was viable, survival was similar between reposition (detorsion) and direct resection, whereas loss of viability favored direct resection.^1^

As MICS becomes more widely adopted, awareness that lung torsion can occur is important. Early diagnosis and prompt surgical intervention are critical to preserve viable lung lobes and to avoid life-threatening complications.

## References

[1] Dai J., Xie D., Wang H. (2016). Predictors of survival in lung torsion: a systematic review and pooled analysis. J Thorac Cardiovasc Surg.

[2] Cable D.G., Deschamps C., Allen M.S. (2001). Lobar torsion after pulmonary resection: presentation and outcome. J Thorac Cardiovasc Surg.

[3] Zhu J., Zhang Y., Bao C., Ding F., Mei J. (2022). Individualized strategy of minimally invasive cardiac surgery in congenital cardiac septal defects. J Cardiothorac Surg.

[4] Apostolakis E., Koletsis E.N., Panagopoulos N., Prokakis C., Dougenis D. (2006). Fatal stroke after completion pneumonectomy for torsion of left upper lobe following left lower lobectomy. J Cardiothorac Surg.

[5] Kucich V.A., Villarreal J.R., Schwartz D.B. (1989). Left upper lobe torsion following lower lobe resection. Early recognition of a rare complication. Chest.

[6] Irie M., Okumura N., Nakano J. (2014). Spontaneous whole-lung torsion after massive pleural effusion and atelectasis. Ann Thorac Surg.

[7] Oliveira C., Zamakhshary M., Abdallah M.R. (2007). Lung torsion after tracheoesophageal fistula repair: a case report and review of literature. J Pediatr Surg.

[8] Banki F., Velmahos G.C. (2005). Partial pulmonary torsion after thoracotomy without pulmonary resection. J Trauma.

